# Extensive mucocutaneous, oesophageal and otic lichen planus secondary to nivolumab therapy

**DOI:** 10.1002/ski2.8

**Published:** 2020-11-20

**Authors:** L. Ferguson, B. Ho, J. Weir, N. Francis, K. West, B. Rathbone, J. Larkin, K. Heelan

**Affiliations:** ^1^ Royal Marsden Hospital NHS Trust London UK; ^2^ Dermatology Department St George's University Hospitals NHS Foundation Trust London UK; ^3^ Department of Histopathology Imperial College Healthcare NHS Trust London UK; ^4^ Histopathology Department Leicester Royal Infirmary Leicester UK; ^5^ Gastroenterology Department Leicester Royal Infirmary Leicester UK

## Abstract

We report a 73‐year‐old female with metastatic renal cell carcinoma who developed a widespread lichenoid reaction following nivolumab treatment. The timeline of the reaction strongly correlated with the nivolumab treatment and subsequent cessation. Our patient had cutaneous, mucosal, otic, ophthalmic and oesophageal involvement, demonstrating the potentially extensive nature of lichenoid reactions to anti‐programmed cell death receptor‐1 (anti‐PD1) therapies. Although lichenoid reactions to anti‐PD1 therapies are now well recognized, there have been no previous reports of otic or oesophageal involvement in the literature. Although cutaneous lichenoid reactions do not tend to be severe or treatment limiting, more widespread systemic lichenoid reactions are challenging to manage, particularly in the context of malignancy. This very unusual case highlights the importance of considering involvement beyond the skin in all lichenoid skin reactions.

1

**What's already known about this topic?**

- Lichenoid reactions to anti‐PD1 therapies are now well recognized
- Widespread systemic lichenoid reactions are more challenging to manage

**What does this study add?**

- No previous reports of otic or oesophageal involvement in the literature

## 2 CASE REPORT

We report an extensive lichenoid reaction associated with nivolumab therapy. Following a left humerus pathological fracture, a 73‐year old female was diagnosed with metastatic renal clear cell carcinoma. The fracture site was resected and treated with radiotherapy. Whole‐body magnetic resonance imaging identified bony lesions and computerised tomography staging at 3 months revealed disease progression with new bone involvement. Initial management with tyrosine kinase inhibitor (sunitinib) was complicated by diarrhoea and cardiac failure necessitating discontinuation. We started her on nivolumab, a programmed cell death receptor‐1 (PD‐1) inhibitor.

By inhibiting PD‐1, nivolumab facilitates T‐cell activation, which potentiates an immune response to tumour cells and is recommended for treatment of several malignancies. Excess T‐cell activation may induce T‐cell‐mediated diseases. Most immune‐related adverse events (irAEs) improve or resolve with corticosteroids and treatment modifications; however, life‐threatening irAEs have been reported.

After the initiation of nivolumab, a good response was noted on imaging over 12 months. She developed irAEs including arthritis, mild colitis and pneumonitis at 6 months. Grade 3 colitis at 12 months required intravenous methylprednisolone followed by prednisolone 1 mg/kg and temporary cessation of nivolumab.

**FIGURE 1:**
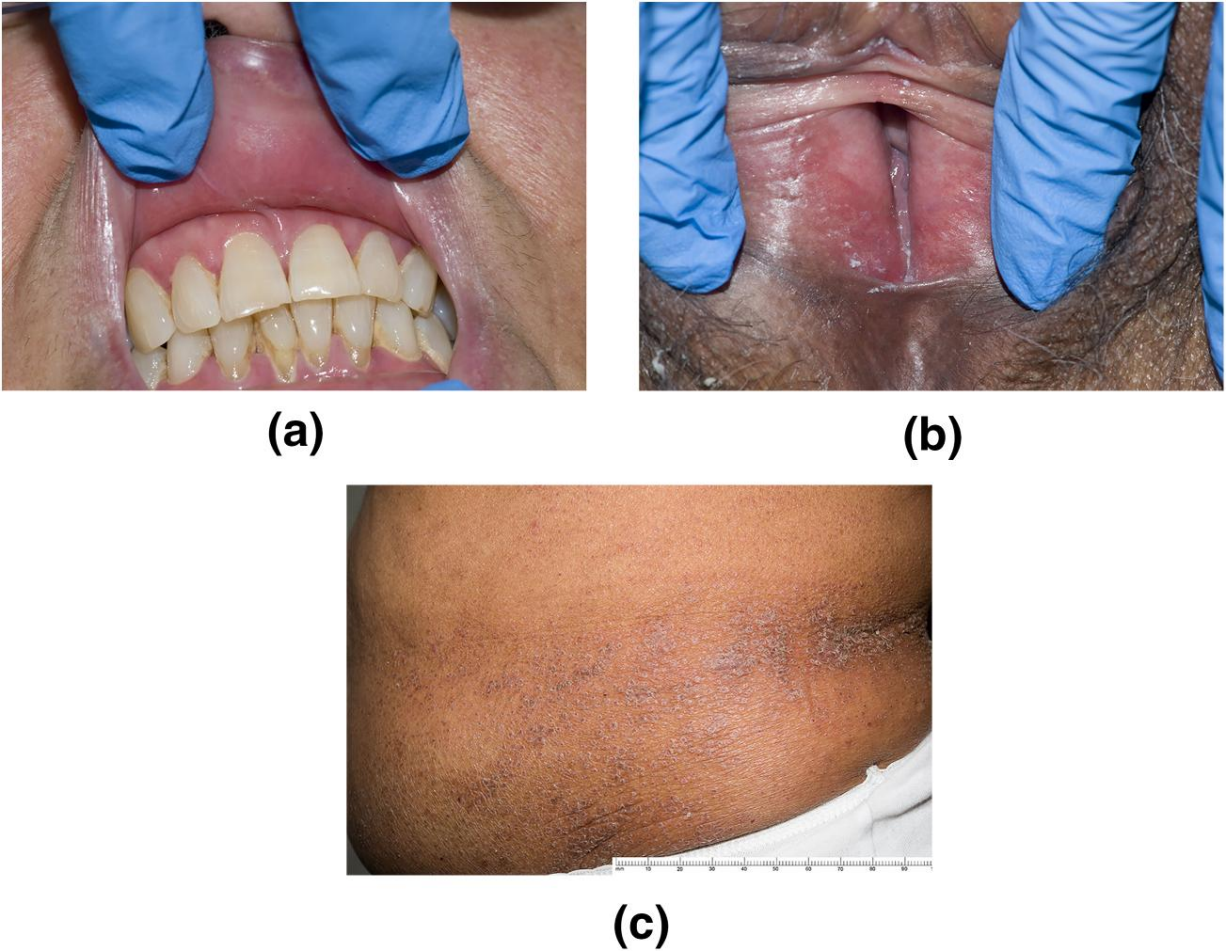
(a) Apical erythema of the gingiva. (b) Symmetrical tender erythema of the labia. (c) A pruritic rash of the flank and lower abdomen consisting of flat‐topped purple papules

**FIGURE 2:**
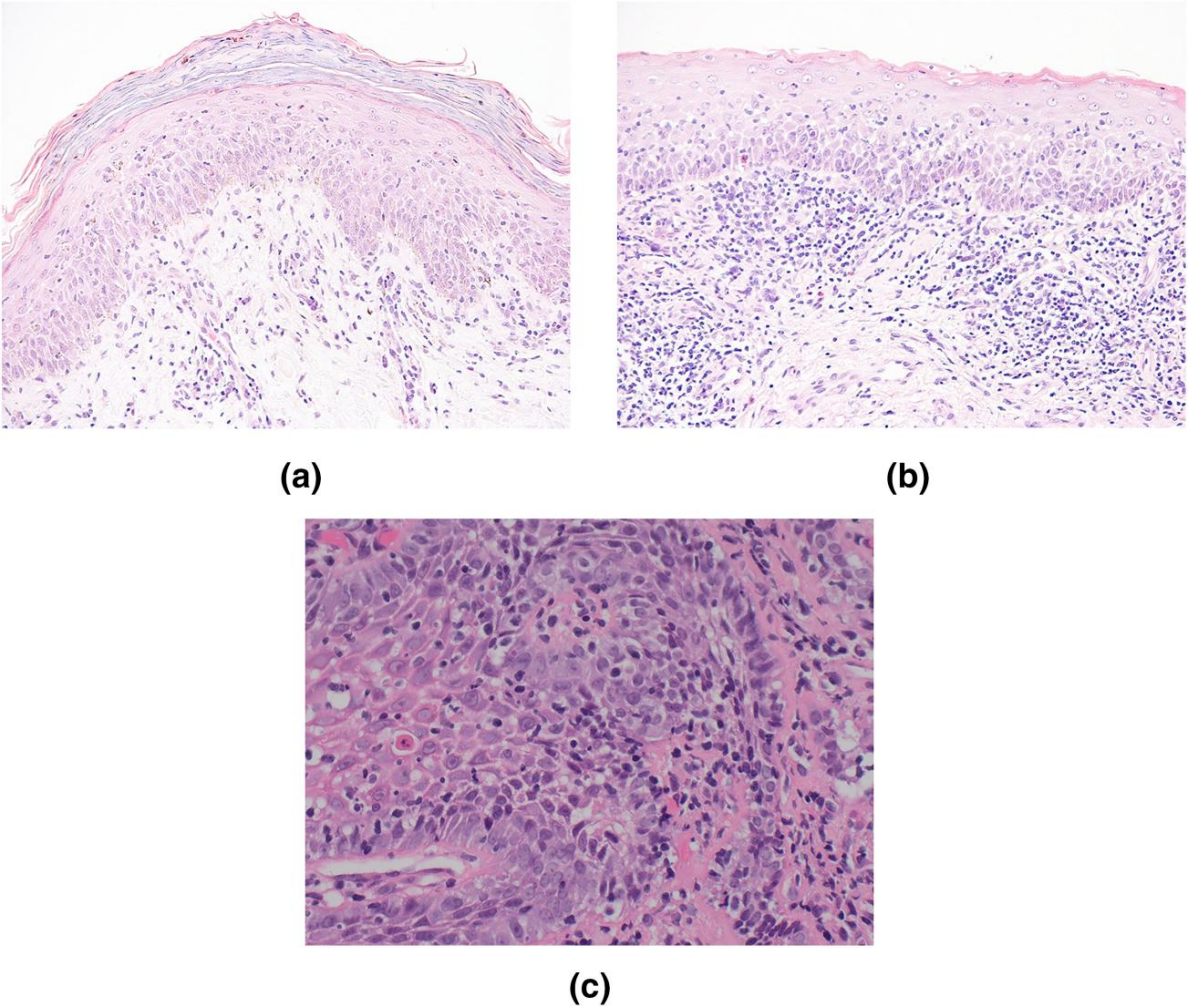
(a) Skin biopsy from the abdomen (×200 magnification) showed classic features of lichenoid inflammation with dyskeratotic keratinocytes and some eosinophils (not shown) consistent with a drug‐induced lichenoid reaction. (b) Skin biopsy of the vulva (×200 magnification) demonstrating parakeratosis, a dense lichenoid lymphohistiocytic infiltrate, mild basal degeneration and colloid bodies within the epidermis, consistent with lichenoid inflammation. (c) Oesophageal biopsy (×40 magnification) showing squamous mucosa with chronic inflammation concentrated around basal epithelium and Civatte body formation consistent with lichen planus

Eighteen months after commencing nivolumab, she developed vulval pain, painful oral ulceration, dysphagia, a widespread pruritic rash and weight loss. Examination of the oral mucosa revealed Wickham's striae and apical erythema of the gingiva (Figure 1a). Genital examination demonstrated symmetrical severe erythema and tenderness of the labia minora (Figure 1b). A widespread purple, papular rash affecting the arms, back, flanks and lower abdomen is shown (Figure 1c). A skin biopsy from the abdomen showed lichenoid inflammation with dyskeratotic keratinocytes and eosinophils, consistent with a lichenoid reaction (Figure 2a).

Cutaneous adverse events (AEs) to PD‐1 inhibitors, including lichen planus (LP), are increasingly reported.^1^ The incidence of cutaneous AE varies (8.7%^1^–49%^2^). Incidence of irAEs increases with duration of treatment,^2^ but the onset of LP after treatment initiation varies (2 weeks^3^ to months). LP, a T‐cell‐mediated chronic inflammatory disease, may be a new phenomenon, or represent a recurrence of quiescent LP.^3^ Classic LP is commonly described, but hypertrophic^3^ and bullous LP^4^ are also reported.

In our patient, initial treatment with super‐potent topical steroids and betamethasone mouthwashes yielded a partial response. A subsequent flare of colitis necessitated the use of systemic steroids and the cessation of nivolumab. This led to a resolution of both her rash and mucositis. As prednisolone was weaned, the gingival and vulvovaginal symptoms recurred. A vulval biopsy confirmed a lichenoid reaction (Figure 2b) and demonstrated a negative direct immunofluorescence. Dysphagia and hoarseness developed. Oesophageal biopsies demonstrated inflammation of the squamous mucosa consistent with LP (Figure 2c). A slow wean of oral prednisolone 0.5 mg/kg ensued with continued topical corticosteroid application. Current maintenance is with low‐dose acitretin (10 mg daily), topical corticosteroids as required and fluticasone propionate pharyngeal spray. Recent development of aural symptoms (scaling, debris, pruritis and otorrhoea) due to lichenoid inflammation of the external auditory canal improved with topical tacrolimus drops. Ophthalmological symptoms (punctual occlusion) required hot compress, massage and topical steroids.

Idiopathic LP may affect skin or mucous membranes including vagina, vulva, oral cavity, ear canals, oesophagus and lacrimal ducts. Anti‐PD‐1‐induced mucous‐membrane LP was considered rare (1/14 patients),^2^ but recent case series suggest oral reactions are under‐recognized or asymptomatic.^5^ One series has described anti‐PD‐1‐induced genital LP (in 2/10 patients with oral reactions).^5^ Oesophageal LP is a rare manifestation of idiopathic LP.^6^ Radiotherapy may trigger LP at radiation sites and radiation may potentiate the autoimmune toxicity of nivolumab.^1^, ^3^

This case demonstrates the management challenge of systemic lichenoid reactions, in the context of malignancy. Cutaneous LP is usually managed with topical treatment. Vulvovaginal, oesophageal and aural LP often require systemic treatment, interruption of anti‐PD‐1 therapy and intervention including endoscopic dilatations.^6^

To our knowledge, oesophageal and aural LP have not been reported in anti‐PD‐1‐induced LP. In patients with cutaneous LP, involvement of widespread mucosal sites should be considered.

## CONFLICT OF INTERESTS

No conflict of interests have been declared.

## References

[1] Hofmann L , Forschner A , Loquai C , et al. Cutaneous, gastrointestinal, hepatic, endocrine, and renal side‐effects of anti‐PD‐1 therapy. Eur J Cancer. 2016;60:190–209. 27085692 10.1016/j.ejca.2016.02.025

[2] Hwang SJ , Carlos G , Wakade D , et al. Cutaneous adverse events (AEs) of anti‐programmed cell death (PD)‐1 therapy in patients with metastatic melanoma: a single‐institution cohort. J Am Acad Dermatol. 2016;74(3):455–61.e1. 26793994 10.1016/j.jaad.2015.10.029

[3] Maarouf M , Alexander C , Shi VY . Nivolumab reactivation of hypertrophic lichen planus, a case report and review of published literature. Dermatol Online J. 2018;24(1):9. 29469765

[4] Biolo G , Caroppo F , Salmaso R , Alaibac M . Linear bullous lichen planus associated with nivolumab. Clin Exp Dermatol. 2019;44(1):67–8. 29920744 10.1111/ced.13700

[5] Sibaud V , Eid C , Belum VR , et al. Oral lichenoid reactions associated with anti‐PD‐1/PD‐L1 therapies: clinicopathological findings. J Eur Acad Dermatol Venereol. 2017;31(10):e464–9. 10.1111/jdv.14284 PMC5645209 28419570

[6] Mohammed AR , Sherwood P . Oesophageal lichen planus: a missed diagnosis. J R Soc Med. 2006;99(6):318–20. 16738377 10.1258/jrsm.99.6.318 PMC1472714

